# Interaction of schizophrenia and chronic cannabis use on reward anticipation sensitivity

**DOI:** 10.1038/s41537-021-00163-2

**Published:** 2021-06-16

**Authors:** Simon Fish, Foteini Christidi, Efstratios Karavasilis, Georgios Velonakis, Nikolaos Kelekis, Christoph Klein, Nicholas C. Stefanis, Nikolaos Smyrnis

**Affiliations:** 1 Laboratory of Cognitive Neuroscience and Sensorimotor Control, University Mental Health, Neurosciences and Precision Medicine Research Institute “COSTAS STEFANIS”, Athens, Greece; 2 1st Department of Psychiatry, National and Kapodistrian University of Athens, School of Medicine, Eginition Hospital, Athens, Greece; 3 Department of Medical Physics, National and Kapodistrian University of Athens, School of Medicine, Athens, Greece; 4 2nd Department of Radiology, National and Kapodistrian University of Athens, School of Medicine, University General Hospital “ATTIKON”, Athens, Greece; 5 2nd Department of Psychiatry, National and Kapodistrian University of Athens, School of Medicine, University General Hospital “ATTIKON”, Athens, Greece; 6 Department of Child and Adolescent Psychiatry, Medical Faculty, University of Freiburg, Freiburg, Germany; 7 Department of Child and Adolescent Psychiatry, Medical Faculty, University of Cologne, Cologne, Germany

**Keywords:** Schizophrenia, Neuroscience

## Abstract

Chronic cannabis use and schizophrenia are both thought to affect reward processing. While behavioural and neural effects on reward processing have been investigated in both conditions, their interaction has not been studied, although chronic cannabis use is common among these patients. In the present study eighty-nine participants divided into four groups (control chronic cannabis users and non-users; schizophrenia patient cannabis users and non-users) performed a two-choice decision task, preceded by monetary cues (high/low reward/punishment or neutral), while being scanned using functional magnetic resonance imaging. Reward and punishment anticipation resulted in activation of regions of interest including the thalamus, striatum, amygdala and insula. Chronic cannabis use and schizophrenia had opposing effects on reward anticipation sensitivity. More specifically control users and patient non-users showed faster behavioural responses and increased activity in anterior/posterior insula for high magnitude cues compared to control non-users and patient users. The same interaction pattern was observed in the activation of the right thalamus for reward versus punishment cues. This study provided evidence for interaction of chronic cannabis use and schizophrenia on reward processing and highlights the need for future research addressing the significance of this interaction for the pathophysiology of these conditions and its clinical consequences.

## Introduction

The chronic use of cannabis increases the risk of developing schizophrenia^1^. This risk increases with rising total exposure to cannabis^2^. Chronic cannabis use has been associated with younger age of psychosis onset and there is evidence of a positive correlation between age of chronic use onset and age of psychosis onset^3^. Furthermore, a younger age of psychosis onset has been associated with chronic use of high-potency cannabis on a daily basis^3^.

The incidence of chronic cannabis use is greater in patients with schizophrenia compared to the general population^4^. Chronic cannabis user patients have a higher risk of psychotic relapse, more hospital admissions and a higher duration of hospital stay, as well as increased usage of antipsychotic medication^5^. On the other hand, it has been shown that chronic cannabis-using patients perform better than non-using patients in cognitive tests^6–8^. At the neural level patients who use cannabis have been shown to display differences in functional brain activation compared to non-user patients in a variety of domains including emotional memory and visuospatial tasks^9,10^.

Differences in reward processing have been demonstrated in both chronic cannabis users and schizophrenia patients. Some studies have shown that chronic users of cannabis have reduced sensitivity to non-drug-related rewards^11^. The effects of reward on cognitive processing have been studied using variations of the monetary incentive delay (MID) task in which reward and/or punishment anticipating cues are followed by a delayed response^12–15^. Using the MID task, studies have reported no reward-related differences in reaction time (RT) amongst users and non-users^11,16–19^. Some studies have reported hypersensitivity in the striatum while anticipating reward and punishment, a reflection of a hypersensitive mesolimbic reward system response to all types of reward in chronic cannabis users^16^. It is not known whether the use of cannabis induces this hypersensitivity or whether it is inherent in some individuals, driving them to seek out cannabis and other types of reward^16^. However, other studies have shown cannabis use to have no effect on neural response to reward and punishment anticipation^17,18^ and yet another study showed hypo-activation in some regions, e.g., the caudate^11^. Differential activation patterns of valence type have also been reported, with cannabis users displaying an increase in ventral striatal activation for reward compared to punishment, while healthy controls exhibited the opposite effect^19^.

Some studies using the MID task in schizophrenia have reported smaller differences in RT for incentive than non-incentive trials in patients compared to controls^20,21^, however others have reported no group differences^22–24^. At the neural level, some studies showed hypo-activation of reward-related brain regions during anticipation of reward^25,26^. Such hypo-activation has been observed in antipsychotic naïve individuals and those treated with typical antipsychotics but has been shown to normalise in those treated with atypical antipsychotics^24,27,28^. Studies have also reported a reduction in striatal activation to be associated with negative symptomatology^20–23,29^.

To the best of our knowledge, the combined effects of chronic cannabis use and schizophrenia on reward-related behaviour and functional brain activation have not been studied. While one MID study compared antipsychotic naïve schizophrenia patients with previous or ongoing substance abuse with non-using counterparts, this was not specific to cannabis and the effects of substance use were not the main focus of the study^27^.

In the present study, we used a two-choice RT task^30^ combined with the MID task to study behavioural and neural responses to anticipated reward and punishment in schizophrenia patients and healthy controls, both with and without a history of chronic cannabis use. Reward anticipation sensitivity effects were measured both behaviourally via changes in RT and accuracy as well as neurally via changes in the activity of reward-related brain areas, with the amount of anticipated reward or punishment. Based on the hypothesis that cannabis sensitizes the reward system of the brain^16^ it was expected that chronic cannabis use would result in increased reward-related sensitivity both at the behavioural and neural level in control chronic cannabis users. Based on previous studies we also expected to find no effect in reward sensitivity for schizophrenia patients when considered as a homogenous group. We further hypothesized that this net effect could be the result of hyposensitivity related to the effects of schizophrenia in non-user patients and hypersensitivity related to chronic cannabis use in chronic cannabis user patients.

## Results

### Demographics

Demographic information for the eighty-three participants included in the behavioural analysis, including cannabis use data is presented in Table 1. The pattern of use was gathered via self-report measures. Non-cannabis user schizophrenia patients (SZ−C) and cannabis user schizophrenia patients (SZ + C) did not differ in total duration of the disorder, the number of hospitalisations nor medication dosage. Cannabis user healthy controls (HC + C) and SZ + C did not differ in lifetime use, nor duration, frequency or age of first use. Minimum lifetime usage for cannabis users (HC + C and SZ + C) users was 208 times, and maximum lifetime usage for non-cannabis users (HC−C and SZ−C) was 15 times. There were no sex differences among the four groups but the effect of age approached significance (*F*_3,79_ = 2.63, *p* = 0.056, η_p_^2^ = 0.091). Participants differed significantly in years of education (*F*_3,79_ = 12.41, *p* < 0.0001, η_p_^2^ = 0.32). Age and education level were included as continuous covariates in all analyses including group effects.

**Table 1:** Demographic data for the eighty-three participants that were included in the behavioural analysis.

Measure	HC−C (*n* = 27)	HC + C (*n* = 22)	SZ-C (n = 21)	SZ + C (*n* = 13)	*p*
Age (years)	27.82 (4.63)	27.05 (7.72)	30.29 (8.00)	23.92 (4.75)	0.056^a^
Sex (% male)	63	77	81	92	0.16^b^
Education level (years)	15.63 (0.79)	14.64 (1.68)	12.76 (1.86)	13.46 (1.66)	**<0.0001**^a^
Clinical data					
Chlorpromazine equivalent (mg)			522 (410)	829 (538)	0.09^c^
Disorder duration (years)			3.50 (4.18)	1.62 (1.93)	0.13^c^
Number of hospitalizations			1.44 (0.94)	1.09 (0.54)	0.32^c^
Cannabis use					
Lifetime use (times used)	3.2 (5.4)	3443.8 (4949)	0.8 (1.2)	3488.3 (4896)	0.98^c^
Duration of use (years)		7.78 (5.42)		6.67 (4.16)	0.54^c^
Frequency of use (per week)		6.45 (4.16)		8.58 (8.44)	0.33^c^
Age of first use (years)		16.91 (2.09)		15.46 (2.22)	0.06^c^

Duration, frequency and age of first use for HC−C and SZ−C were not reported since most of them did not use cannabis. Lifetime use is an estimation based on duration and frequency of use. All measures apart from sex are equivalent to the mean of the respective group. Parentheses indicate standard deviation.

Bold typeface = *p* < 0.05. *p* values for all cannabis use variables indicate significance for testing differences between HC + C and SZ + C.

*HC−C* non-cannabis user healthy controls, *HC + C* cannabis-user healthy controls, *SZ−C* non-cannabis user schizophrenia patients, *SZ + C* cannabis-user schizophrenia patients.

^a^Analysis of variance (ANOVA) was used.

^b^Chi-square test was used.

^c^Independent samples *t*-test was used.

### Behavioural global analysis

There was no significant effect of reward on directional accuracy (DA) (*F*_4,308_ = 1.78, *p* = 0.132, η_p_^2^ = 0.022). There was no significant interaction of reward x cannabis use (*F*_4,308_ = 1.54, *p* = 0.19, η_p_^2^ = 0.019), no significant interaction of reward x schizophrenia (*F*_4,308_ = 1.6, *p* = 0.174, η_p_^2^ = 0.02) and no significant three-way interaction of reward x cannabis x schizophrenia (*F*_4,308_ = 2.04, *p* = 0.088, η_p_^2^ = 0.026) on DA.

The effect of reward on RT was not significant (*F*_4,308_ = 0.86, *p* = 0.485, η_p_^2^ = 0.011) and there was no significant interaction of reward × cannabis use (*F*_4,308_ = 0.66, *p* = 0.617, η_p_^2^ = 0.008) nor reward × schizophrenia (*F*_4,308_ = 0.61, *p* = 0.659, η_p_^2^ = 0.008). There was however a highly significant three-way interaction of reward x cannabis x schizophrenia (*F*_4,308_ = 3.05, *p* = 0.017, η_p_^2^ = 0.038) on RT. The global analysis was also performed on the seventy-three individuals that were retained in the imaging analysis and the results were similar (not presented).

### Behavioural contrast analysis

**Fig. 1:**
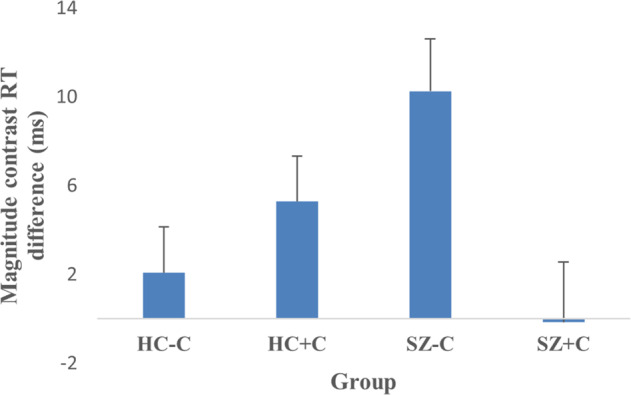
Reaction time (RT) differences between groups. Mean RT difference (ms) for magnitude contrast for each group. ms milliseconds, HC−C non-cannabis user healthy controls, HC + C cannabis-user healthy controls, SZ−C non-cannabis user schizophrenia patients, SZ + C cannabis-user schizophrenia patients. Error bars indicate standard errors of the mean differences.

Results from the global analysis revealed significant interactions only for RT. For this reason, only this measure was further investigated in the contrast analysis. The valence contrast was not modulated by cannabis use (*F*_1,77_ = 1.23, *p* = 0.27, η_p_^2^ = 0.016), neither by schizophrenia (*F*_1,77_ = 0.19, *p* = 0.66, η_p_^2^ = 0.002), nor their interaction (*F*_1,77_ = 0.53, *p* = 0.47, η_p_^2^ = 0.007). The reward versus punishment contrast was not modulated by cannabis use (*F*_1,77_ = 0.002, *p* = 0.97, η_p_^2^ = 0.0002) nor by schizophrenia (*F*_1,77_ = 1.4, *p* = 0.24, η_p_^2^ = 0.018) but was significantly modulated by their interaction (*F*_1,77_ = 4.57, *p* = 0.036, η_p_^2^ = 0.056). This effect was however not retained when using the 73 individuals of the imaging sample (*F*_1,67_ = 2.98, *p* = 0.088, η_p_^2^ = 0.042). Finally the magnitude contrast was not significantly modulated by cannabis use (*F*_1,77_ = 2.74, *p* = 0.10, η_p_^2^ = 0.033) nor schizophrenia (*F*_1,77_ = 0.27, *p* = 0.60, η_p_^2^ = 0.003) but was significantly modulated by their interaction (*F*_1,77_ = 7.64, *p* = 0.007, η_p_^2^ = 0.09). This effect was also retained when using the seventy-three individuals of the imaging sample (*F*_1,67_ = 8.86, *p* = 0.004, η_p_^2^ = 0.117). Figure 1 shows that the magnitude contrast in RT (corresponding to an increase in speed for the high reward and punishment magnitude cues compared to low magnitude and neutral cues) was larger in HC + C compared to HC−C, while the opposite effect was observed for schizophrenia patients, namely a decrease for SZ + C compared to SZ−C.

### Imaging validation analysis

**Fig. 2:**
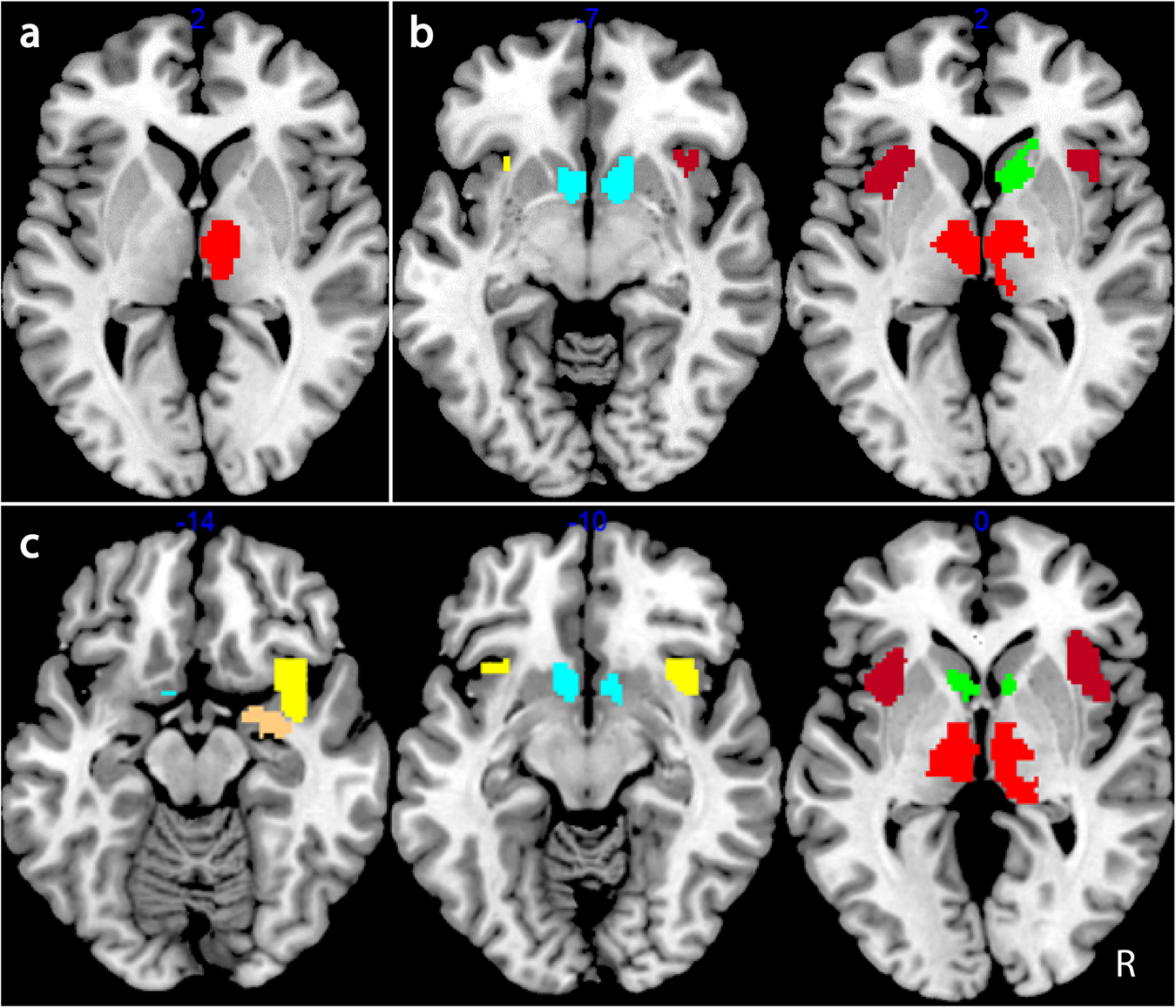
Validation analysis. Clusters of higher activation for reward + punishment versus neutral conditions for the valence (**a**) and magnitude (**b**) cue period as well as high versus low + neutral for the magnitude cue period (**c**). Clusters thresholded at *p* < 0.005 for visualisation purposes. Red = thalamus; green = caudate; cyan = nucleus accumbens; peach = amygdala; brown = dorsal anterior insula; yellow = ventral anterior insula.

Table 2 and Fig. 2 present the results of the validation analysis. One-sample *t*-tests across all subjects confirmed that reward-related regions, assessed by region of interest (ROI) analysis, were more highly activated in both the valence and magnitude models, for the valence contrast with right thalamus being more highly activated for incentive conditions compared to neutral during the valence cue period. Additional regions were significantly more highly activated for incentive compared to neutral conditions during the presentation of the magnitude cue including the left: thalamus and ventral anterior insula, right: caudate, as well as bilateral: dorsal anterior insula and nucleus accumbens (NAcc). The magnitude contrast revealed high magnitude cues compared to low magnitude plus neutral ones further activated the right: ventral anterior insula and amygdala and left: caudate. There were no differences in activation for the reward versus punishment contrast in any pre-defined ROI.

**Table 2:** Region of interest (ROI) validation analysis for the three contrasts using the valence and magnitude cue models.

Contrast	Anatomical labelling			Statistics		MNI coordinates			
	Label	Hemisphere	*Z*	p(svc)	*K*_E_	*x*	*y*		z
Reward + Punishment > Neutral									
Valence model	Thalamus	R	3.84	0.003	20	9	−7		−2
Magnitude model	dAI	L	4.24	0.001	38	−33	23		−2
		R	3.39	0.013	17	42	17		−2
	vAI	L	3.65	0.006	3	−30	20		−5
	NAcc	L	3.33	0.015	5	−3	8		−5
		R	4.60	0.000	25	9	5		−5
	Caudate	R	4.42	0.000	16	9	5		−2
		R	3.28	0.017	3	18	26		1
	Thalamus	L	3.73	0.004	27	−6	−10		−2
		R	3.79	0.003	31	3	−10		1
Reward > Punishment									
Valence model	–	–	–	–	–	–	–		–
Magnitude model	–	–	–	–	–	–	–		–
High > Low + Neutral									
Magnitude model	Caudate	L	4.36	0.000	17	−6	8		−2
		R	4.11	0.001	14	9	5		−2
	NAcc	L	4.26	0.001	29	−6	8		−5
		R	4.38	0.000	12	9	5		−5
	Amygdala	R	4.18	0.001	12	18	−1		−17
	Thalamus	L	3.82	0.003	62	−15	−10		10
		R	3.94	0.002	39	6	−4		4
	dAI	L	3.87	0.002	23	−33	23		−5
		R	4.57	0.000	43	33	23		−8
	vAI	L	3.78	0.003	15	−36	17		−5
		R	4.71	0.000	35	30	20		−11

We applied family-wise error (FWE) correction adjusted for small volume [*p* (svc) < 0.05] within each of the independent ROIs at the voxel level (only ROIs with at least three contiguous voxels were considered significant). There were no significantly different regions for the reward vs punishment contrast.

*MNI* Montreal Neurological Institute, *svc* small-volume correction, *R* right, L left, *K*_*E*_ number of voxels in cluster, *dAI* dorsal anterior insula, *vAI* ventral anterior insula, *NAcc* nucleus accumbens.

### Imaging main analysis

**Fig. 3:**
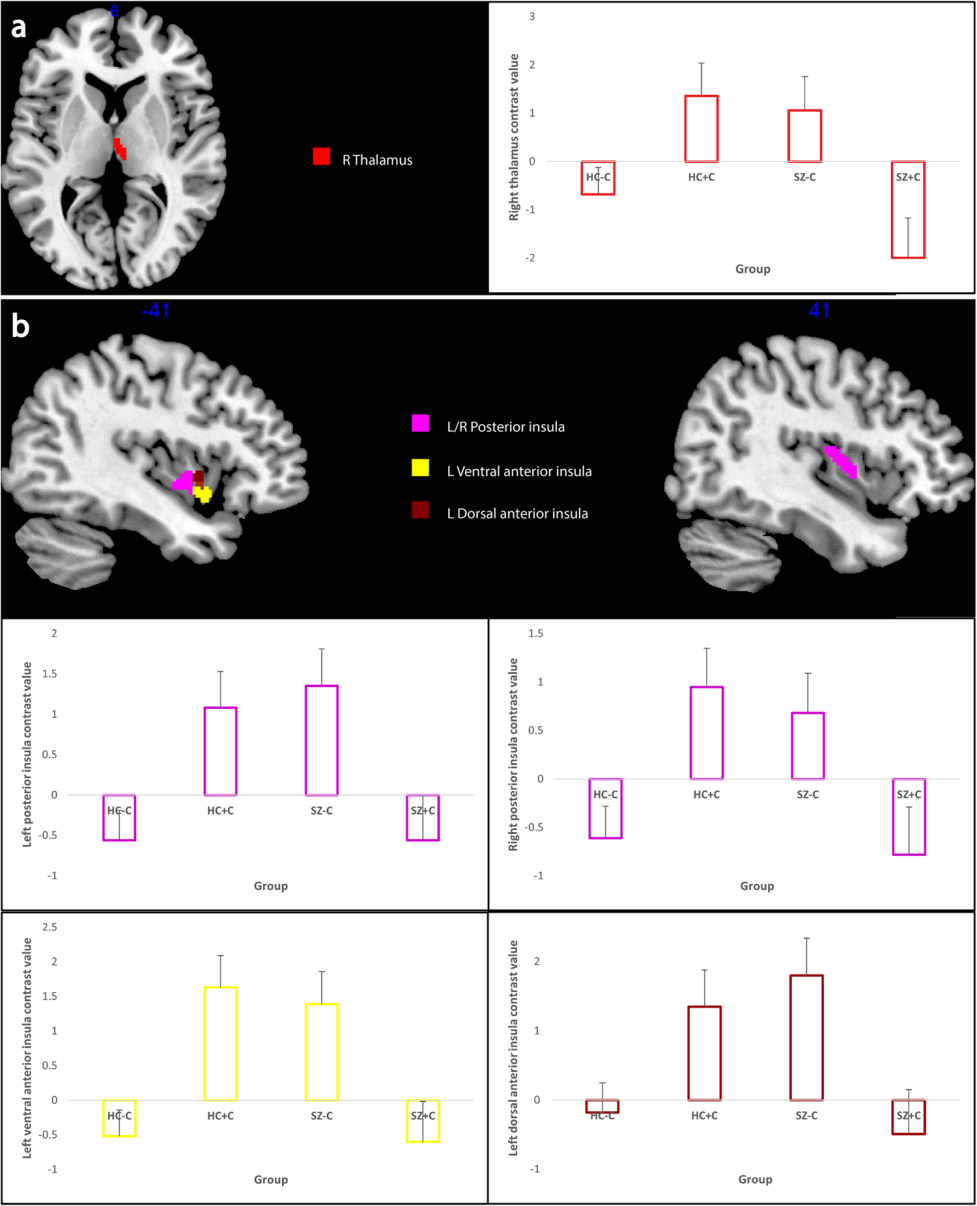
Main analysis. Clusters showing significant modulation by the interaction of cannabis and schizophrenia for the reward versus punishment contrast (**a**) and the magnitude contrast (**b**). Clusters thresholded at *p* < 0.005 for visualisation purposes. Red = thalamus; yellow = ventral anterior insula; brown = dorsal anterior insula; violet = posterior insula. The bar plots show mean beta values for each cluster for each group and error bars show standard errors of the mean beta values. HC−C non-cannabis user healthy controls, HC + C cannabis-user healthy controls, SZ−C non-cannabis user schizophrenia patients, SZ + C cannabis-user schizophrenia patients.

Based on the results of the validation analysis, the main analysis was carried out on the contrasts for the magnitude model. Between-subjects results of the main analysis including the effects of cannabis, schizophrenia and their interaction are presented in Table 3. There were no between group differences, nor interaction for the valence contrast. The main effect of cannabis use and an interaction of cannabis use and schizophrenia was observed for the reward versus punishment contrast. Extraction of beta values showed an increased activation in the right: putamen, ventral anterior insula and dorsal anterior insula for reward versus punishment for cannabis users (HC + C and SZ + C) compared to non-users (HC−C and SZ−C). Also activation in the right thalamus was larger for reward versus punishment for the HC + C and SZ−C groups versus HC−C and SZ + C groups (Fig. 3a). For the magnitude contrast, there was no main effect of cannabis use nor schizophrenia while the interaction of these two factors appeared for left: ventral anterior insula, dorsal anterior insula and bilateral posterior insula. Following beta value extraction it was shown that HC + C exhibited increased activation in each of the above-mentioned regions compared to HC−C, while the opposite pattern was observed for patients, namely SZ + C displayed activation decreases in all of these regions compared to SZ−C (Fig. 3b).

**Table 3:** Region of interest (ROI) main analysis displaying the effects of cannabis and schizophrenia on each contrast of interest as well as the cannabis by schizophrenia interactions.

Contrast	Anatomical labelling			Statistics		MNI coordinates		
	Label	Hemisphere	*F*	*p* (svc)	*K*_E_	*x*	*y*	*z*
Reward + Punishment > Neutral								
Cannabis	–	–	–	–	–	–	–	–
Diagnosis	–	–	–	–	–	–	–	–
Interaction	–	–	–	–	–	–	–	–
Reward > Punishment								
Cannabis	Putamen	R	15.89	0.009	4	24	5	−2
	vAI	R	14.09	0.017	8	39	14	−8
	dAI	R	14.42	0.015	6	39	17	−8
Diagnosis	–	–	–	–	–	–	–	–
Interaction	Thalamus	R	14.48	0.015	5	3	−19	7
High > Low + Neutral								
Cannabis	–	–	–	–	–	–	–	–
Diagnosis	–	–	–	–	–	–	–	–
Interaction	vAI	L	15.93	0.008	12	−39	−1	−5
	dAI	L	14.46	0.014	3	−39	2	−2
	pI	R	22.81	0.001	19	42	−10	13
		L	16.05	0.008	16	−42	2	−8

We applied family-wise error (FWE) correction adjusted for small volume [*p* (svc) < 0.05] within each of the independent ROIs at the voxel level (only ROIs with at least three contiguous voxels were considered significant).

*MNI* Montreal Neurological Institute, *svc* small-volume correction, *R* right, *L* left, *vAI* ventral anterior insula, *dAI* dorsal anterior insula, *pI* posterior insula.

## Discussion

This study investigated the effects of chronic cannabis use and schizophrenia on behaviour and neural activation related to the anticipation of reward and punishment in a two-choice RT task.

There was no overall modulation of DA by reward and punishment and there was no effect on reward-related DA sensitivity of cannabis nor schizophrenia nor their interaction. There was also no overall modulation of RT by reward and punishment and there was no effect on reward-related RT sensitivity of cannabis nor schizophrenia. There was however a significant interaction of cannabis use and schizophrenia. When comparing cannabis users (HC + C and SZ + C) with non-users (HC−C and SZ−C) and schizophrenia patients (SZ−C and SZ + C) with healthy controls (HC−C and HC + C) there was no difference in the sensitivity for high magnitude cues as reflected in the reduction of RT. A very different picture emerged when we studied the interaction of cannabis use and schizophrenia on the behavioural measure of reward sensitivity. Increased sensitivity to high magnitude cues manifested as an increase in speed (reduction in mean RT) clearly dissociated the different groups. Sensitivity was increased in HC + C and SZ−C compared to HC−C and SZ + C. The increase in reward sensitivity that was observed for control cannabis users versus control non-users is in accordance with our first hypothesis and supports the hypothesis of reward hypersensitivity in chronic cannabis use^16^. In contrast to our second hypothesis non-user schizophrenia patients showed increased reward sensitivity compared to non-user controls. Moreover we observed that chronic cannabis user patients showed a decrease instead of the expected increase in reward-related sensitivity compared to non-user patients. In fact the decrease in reward-related sensitivity related to chronic cannabis use fully compensated the increase observed in the non-user patient group resulting in a net null effect of schizophrenia on reward-related sensitivity which is in accordance with previous studies^22–24^. The important factor to consider here is that all these previous studies did not dissociate cannabis user patients from non-users.

Using a version of the MID task we observed an increase of activation in predefined reward-related ROIs, in thalamus, NAcc, caudate and insula for all incentive cues in line with previous studies^31,32^. We also confirmed that high magnitude cues produced a further activation increase in these areas as well as higher amygdala activation, a further important area in reward anticipation^31,33^

The purpose of the study concerned the modulation of reward-related activation by chronic cannabis use and schizophrenia. There was an activation difference between cannabis users (HC + C and SZ + C) and non-users (HC−C and SZ−C), such that users displayed higher activation in the right: putamen, ventral anterior insula and dorsal anterior insula for reward compared to punishment trials, in accordance with previous research showing increased neural sensitivity to reward over punishment^19^.

A much more interesting picture emerged when considering the interaction of cannabis and schizophrenia on reward-related activation. The increase in activation for high magnitude cues compared to low and neutral ones in left: ventral anterior insula, dorsal anterior insula and bilateral posterior insula was larger in HC + C and SZ−C compared to HC−C and SZ + C replicating the results that were observed behaviourally for reward-related sensitivity. The increase in activation related to reward anticipation for control chronic cannabis users compared to non-users confirms our first hypothesis and is in accordance with the previous research^16^. However in contrast to our second and third hypotheses we observed increased activation for high magnitude cues in non-user patients and a decrease in activation for chronic user schizophrenia patients. These opposing effects compensated for each other so that in the total group of patients there was no difference in reward-related sensitivity when compared to the total group of controls that is in accordance with previous studies of schizophrenia patients receiving atypical antipsychotics^24,25,27^. Again it is important to note here that all of these previous studies have not included chronic cannabis use as a factor in the analysis of reward-related sensitivity in schizophrenia.

The majority of research on the involvement of insula on reward anticipation has focused on the anterior sub-region^31,32^, which has been found to be involved in the assessment of risk for upcoming events^34^. Previous studies have shown functional activation differences of chronic cannabis users^35^ and schizophrenia patients^36^ compared to controls in the anterior insula but the combined effects of both groups on activation of this area were not investigated. In the current study we observed an interaction effect of cannabis use and schizophrenia on reward anticipation-related activation on both anterior and posterior insula. Previous research has suggested that increased activity of the posterior insula during reward anticipation may indicate increased somatosensory arousal^37^. The present study showed a specific increase in activation of the left anterior and bilateral posterior insula in relation to high magnitude cues in HC + C and SZ−C compared to HC−C and SZ + C suggesting a sensitization of these reward anticipation-related areas by chronic cannabis use and schizophrenia that diminished when both factors were present.

In response to valence anticipation, thalamic activation has been found to signify an “alerting” response, converging with insular information to guide action selection in NAcc^38^. In this study we observed an increase in right thalamic activation for reward versus punishment cues in HC + C and SZ−C compared to HC−C and SZ + C. This interaction effect once again suggests a reward-specific sensitization produced by chronic cannabis use and schizophrenia that was reversed when both factors were present.

The striking similarity in the pattern of behavioural and neural effects for the three-way interaction of cannabis, schizophrenia and reward modulation could lead to the hypothesis that the chronic use of cannabis in healthy controls (HC + C) and schizophrenia without a history of cannabis use (SZ−C) both increase sensitivity to reward anticipation compared to healthy control non-users (HC−C) manifested in behaviour (speed of decision processing) and neural activation of reward processing areas. Furthermore the chronic use of cannabis in schizophrenia patients (SZ + C) seems to restore this increased reward sensitivity to levels similar to those observed for control non-users (HC−C). Interestingly, a prior study has shown that the administration of oral cannabis and Δ^9^–tetrahydrocannabinol (THC) to schizophrenia patients, can regulate a general dysconnectivity of the reward circuit^39^ and acute administration of cannabidiol (CBD) has been shown to reduce insular activation during reward anticipation in individuals at clinically high-risk of developing psychosis^40^. CBD has been shown to display neuroprotective properties against the toxic effects of THC^41^ and psychosis complications are also more likely to occur following the chronic use of high-potency cannabis, defined by the higher concentration of THC. Future studies are thus needed to investigate the differential effects of THC and CBD on reward anticipation sensitivity in schizophrenia.

The division of our sample in four sub-groups and the specific criteria for inclusion in each group resulted in a reduced number of participants for each individual group. While we see highly significant effects using this sample, increasing the number of participants within each group could result in the emergence of additional significant effects especially concerning the interaction of cannabis and schizophrenia on activation of reward-related areas.

The current study included patients that were medicated and the vast majority received atypical antipsychotics. Although the difference in behavioural and neural reward sensitivity between the two groups of patients cannot be readily attributed to medication, the interaction of medication with reward sensitivity remains an issue that needs to be addressed in future studies investigating the effect of chronic cannabis use in un-medicated or never medicated patients.

Finally all habitual cannabis use data were collected by way of self-report measures in the current study. Due to the fact that self-reports may not be fully accurate combined with the fact that many participants reported regularly using multiple cannabis varieties, potency data was not included in analysis although it is known that potency of cannabis is an important factor when considering the effect of cannabis on psychosis. Future studies could address cannabis potency as an additional factor modulating the effect of cannabis on reward-related sensitivity in schizophrenia.

This study provides evidence for the complex interaction of chronic cannabis use and schizophrenia on the reward system showing that control chronic cannabis users and patients with no history of cannabis use have increased reward-related sensitivity compared to both heathy control non-users and patient users. These results highlight the importance of chronic cannabis use in the investigation of the reward system in schizophrenia and the need for further research in this specific group of patients.

## Methods

### Participants

Eighty-nine participants completed the study, 40 patients and 49 healthy controls. Patients were recruited from the psychosis unit of the psychiatry department at Eginition Hospital and were diagnosed by trained psychiatrists using the International Statistical Classification of Diseases and Related Health Problems, 10th revision (ICD-10)^42^ criteria. One patient received a diagnosis of psychosis not otherwise specified (F29), 34 were diagnosed with schizophrenia (F20) and five with brief psychotic disorder (F23) that were later diagnosed with schizophrenia at follow-up. Thirty-eight patients received atypical antipsychotics (risperidone, paliperidone, olanzapine, amisulpride, quetiapine, aripiprazole, clozapine) and two patients (one user and one non-user) received typical antipsychotics (haloperidole, trifluoperazine).

Pattern of cannabis use was defined using self-report measures. Sixteen patients were classified as SZ + C and twenty-four as SZ−C. Twenty-two healthy control participants were classified as HC + C and 27 as HC−C. Both HC + C and SZ + C were required to have used cannabis a minimum of once per week for one year, within the past year. There were a total of 38 cannabis users across both groups (HC + C and SZ + C) and 51 non-users (HC−C and SZ−C).

Exclusion criteria for all patients (SZ + C and SZ−C) included diagnosis of neurological, neurodevelopmental or other psychiatric disorders as well as the history of illicit drug use, other than cannabis. Exclusion criteria for healthy controls (HC + C and HC−C) also included current use of prescription medication, history of illicit drug use other than cannabis, and personal or familial history of psychiatric or neurological disorder. Participants were also excluded if they declared having used cannabis in the past 24 h or if they were intoxicated with alcohol. An effort was made to match patients and control participants for age and sex.

At the time of testing all patients (SZ + C and SZ−C) were in a stable phase of disorder (they were not currently experiencing a psychotic episode and positive symptoms were in remission) and treated with antipsychotic medication; no participant received benzodiazepines or beta-blockers on the day of testing. All cannabis users (HC + C and SZ + C) were asked to abstain from using for at least 24 h prior to study completion, and asked again on the day of testing to reduce the likelihood of confounding subacute effects. All participants were presented with a detailed description of the study design to ensure that they fully understood the procedures and gave written informed consent. The study protocol was approved by the ethics committee of Eginition University Hospital and was conducted according to the principles of the Declaration of Helsinki.

### Stimuli and procedure

A two-choice RT task was used with elements of the MID and Eriksen flanker tasks. Participants completed the task in one session to reduce the likelihood of learning effects, while being scanned using functional magnetic resonance imaging (fMRI). The participant held a response pad (Cedrus, California, USA) and was instructed to respond to a series of five arrow heads appearing for a fixed period, with their right or left index finger, in accordance with the pointing direction of the central arrowhead. Only the incongruent configuration of the arrow heads was used (< < > < < or > > < > >). Preceding the stimulus, a valence cue was first presented, for a variable period (0.8, 2.8 or 4.8 s), consisting of either + (win), − (lose) or * (neutral), followed by the magnitude cue representing the amount of the upcoming reward (high: 20, low: 5, or none: 0) that was presented for 1 s. After the 1 s response period, feedback was presented for 1.2 s. The participant was informed that the aim of the task was to gain a maximal amount of points and in order for them to win (+) or avoid losing (–), they must respond both accurately and quickly. The task was divided into 6 blocks of 60 trials with the first block consisting solely of neutral trials, used to generate a baseline mean RT from each participant’s correctly answered trials. On subsequent blocks, the participant completed a trial successfully if they responded with the correct button-press and faster or equal to their mean RT from the first block. These five blocks each contained twelve trials of each condition (high punishment, low punishment, neutral, low reward, high reward).

### Behavioural data acquisition and analysis

DA and RT data were analysed for the five blocks of the reward task. 6 patients (3 SZ−C, 3 SZ + C) were excluded from the behavioural analysis due to a < 70% DA, resulting in a total of eighty-three included participants. DA and RT were recorded for each participant and each condition. We excluded RT < 120 ms, considered as anticipatory responses. Total mean DA and RT were calculated for each condition.

A global analysis was performed for DA and mean RT using the general linear model (GLM) and a 2 × 2 × 5 analysis of covariance (ANCOVA) design. Reward condition was the within-subject repeated measures factor (5 levels) while cannabis use and schizophrenia were between-group fixed factors (2 levels each). Finally education level and age were used as continuous covariates. Since the focus of this study was the interaction of reward effects with cannabis use and schizophrenia we report only the reward-related effects of this analysis and not the main effects of cannabis, schizophrenia and their interaction.

A second analysis was performed to investigate the nature of the significant interaction effects between reward conditions and group factors. Following the same rationale as will be presented subsequently for the analysis of the imaging data we computed three specific contrast values for DA and three for mean RT, for each subject as follows:

- valence: difference between the neutral condition and the mean of all valence conditions
- reward versus punishment: difference between mean of reward and mean of punishment conditions.
- magnitude: difference between the mean of low magnitude plus neutral conditions and the mean of high magnitude conditions.

These contrast values for each subject were used as dependent variables in a GLM 2 × 2 ANCOVA with cannabis use and schizophrenia as fixed factors and years of education and age as continuous covariates.

The GLM tool in Statistica 12 (StatSoft Inc., 1984–2014) was used for all analyses of behavioural data.

### fMRI data acquisition and analysis

Functional MR images were acquired using a Philips Achieva 3.0 Tesla TX MRI scanner using echo-planar imaging with 2 s repetition time (TR), 36 slices and 3 × 3 × 3 mm voxel size. A high-resolution T1 anatomical image with 1 × 1 × 1 mm voxel size was also acquired for each participant. Quality control was performed using ArtRepair software (Center for Interdisciplinary Brain Sciences, Stanford University, USA). Ten participants (1 HC−C, 4 HC + C, 4 SZ−C, 1 SZ + C) were excluded due to low image quality, resulting in a sample of seventy-three participants.

SPM12 toolbox for MATLAB (Wellcome Trust Centre for Neuroimaging, London, UK) was used for all imaging data analysis. Pre-processing was first performed by spatially realigning the raw images and temporal interpolation was completed to correct for delay in slice acquisition. Data with registered motion >3 mm or 1 degree was excluded, in keeping with the general rule for exclusion of data with motion greater than the dimensions of a single voxel^43^. The T1 image was next used to segment the images into grey and white matter and cerebrospinal fluid (CSF). Images were normalized to standard Montreal Neurological Institute (MNI) space and smoothed with an 8 mm full width at half maximum (FWHM) Gaussian kernel. The voxel size and smoothing kernel used in our analysis are in accordance with other studies where similar parameters were included in order to study reward processing regions either using whole-brain analysis^44^ or ROI-based analysis, including predefined reward regions, i.e., ventral striatum and insular segments^45,46^. A high-pass filter of 128 s cut off was applied, to eliminate physiological components such as respiration or heartbeat.

Onset times for each condition were extracted for both valence and magnitude cues, with the relative duration for each specific trial and cue type. A first-level within-subject analysis was carried out for both valence and magnitude separately, whereby a GLM was applied to the images from each participant. Three regressors, reward (+), punishment (−) and neutral (*) were included for the valence model. Five regressors (−20, −5, 0, +5, +20) were included for the magnitude model. Additional regressors included motion correction parameters estimated from the realignment step of the pre-processing. T-contrasts were calculated to measure the contrasts of valence, reward versus punishment and magnitude that were defined as previously described. The valence and reward versus punishment contrasts were calculated in the valence model while all three contrasts were calculated in the magnitude model.

At the second-level, a validation ROI analysis was first carried out to verify that reward-related regions were activated during the two cue periods. One-sample *t*-tests were carried out for each contrast. The following ROIs were selected and included in the present study based on a recent meta-analysis of neural activation in the MID task, reporting activation in common regions for reward and punishment anticipation; striatum, thalamus, amygdala and insula^31^. The striatum was divided into subcomponents of NAcc, caudate and putamen and were defined structurally along with thalamus and amygdala, using the Automated Anatomical Labelling atlas 3 (AAL3). Considering the anatomically and functionally distinct insular sub-regions^47^ and their involvement in reward tasks^37,48^, the insula was divided into sub-regions of dorsal and ventral anterior, as well as posterior. Using mean MNI coordinates from a prior study^47^, the insular sub-regions were manually defined on T1^49^ in order to ensure the inclusion of all anatomically relevant regions and the exclusion of anatomically irrelevant regions. All ROIs were defined in MNI space for both right and left hemispheres and were defined before any data analysis in order to avoid bias^50^. Activation within each ROI was assessed with an inclusive mask; the analyses were restricted to the previous ROIs for which control for multiple comparisons was performed using Gaussian random field (GRF) theory for small volume^51^ which allows for conduct principled correction resorting to the GRF theory within a predefined ROI^52^. Small volume correction (SVC) of sphere with 10 mm radius surrounding the peak voxel was applied within these regions and clusters were considered significant if the family-wise error (FWE) corrected peak *p*-value was significant at *p* < 0.05, as in previous studies^45,53^ A minimum cluster size threshold of three contiguous voxels was considered in all analyses to avoid type-1 errors^54^.

The main analysis was a 2 × 2 ANCOVA to assess the modulation of each contrast with cannabis use, schizophrenia status and their interaction, with years of education and age as covariates. Using Marsbar, beta values for each significant voxel cluster were extracted for each participant to assess the nature of the interaction by means of plots.

### Reporting summary

Further information on research design is available in the Nature Research Reporting Summary linked to this article.

## Supplementary information

Reporting Summary

## Data Availability

All the data presented and analysed in this study are fully available from the authors upon request.
