# Supplementary material for: Interaction of schizophrenia and chronic cannabis use on reward anticipation sensitivity
Source: NPJ Schizophr. 2021 Jun 16;7:33. doi: 10.1038/s41537-021-00163-2 (PMC8209034; doi:10.1038/s41537-021-00163-2)
Supplement: Supplementary file 1 — Reporting Summary [file 41537_2021_163_MOESM1_ESM.pdf]

nature research

Corresponding author(s): Nikolaos Smyrnis

Last updated by author(s): Mar 24, 2021

Reporting Summary

Nature Research wishes to improve the reproducibility of the work that we publish. This form provides structure for consistency and transparency in reporting. For further information on Nature Research policies, see our Editorial Policies and the Editorial Policy Checklist.

Statistics

For all statistical analyses, confirm that the following items are present in the figure legend, table legend, main text, or Methods section.

n/a	Confirmed	
☐	☒	The exact sample size (n) for each experimental group/condition, given as a discrete number and unit of measurement
☐	☒	A statement on whether measurements were taken from distinct samples or whether the same sample was measured repeatedly
☐	☒	The statistical test(s) used AND whether they are one- or two-sided<br>*Only common tests should be described solely by name; describe more complex techniques in the Methods section.*
☐	☒	A description of all covariates tested
☐	☒	A description of any assumptions or corrections, such as tests of normality and adjustment for multiple comparisons
☐	☒	A full description of the statistical parameters including central tendency (e.g. means) or other basic estimates (e.g. regression coefficient) AND variation (e.g. standard deviation) or associated estimates of uncertainty (e.g. confidence intervals)
☐	☒	For null hypothesis testing, the test statistic (e.g. F, t, r) with confidence intervals, effect sizes, degrees of freedom and P value noted<br>*Give P values as exact values whenever suitable.*
☒	☐	For Bayesian analysis, information on the choice of priors and Markov chain Monte Carlo settings
☐	☒	For hierarchical and complex designs, identification of the appropriate level for tests and full reporting of outcomes
☐	☒	Estimates of effect sizes (e.g. Cohen's d, Pearson's r), indicating how they were calculated

Our web collection on statistics for biologists contains articles on many of the points above.

Software and code

Policy information about availability of computer code

Data collection	E-prime version Psychology Software Tools, Philips Achieva 3.0 Tesla TX MRI scanner
Data analysis	Statistica version 12 (StatSoft Inc. 1984-2014), SPM Matlab ToolBox

For manuscripts utilizing custom algorithms or software that are central to the research but not yet described in published literature, software must be made available to editors and reviewers. We strongly encourage code deposition in a community repository (e.g. GitHub). See the Nature Research guidelines for submitting code & software for further information.

Data

Policy information about availability of data

All manuscripts must include a data availability statement. This statement should provide the following information, where applicable:

- Accession codes, unique identifiers, or web links for publicly available datasets
- A list of figures that have associated raw data
- A description of any restrictions on data availability

All data used in this study are available from the authors upon request

Field-specific reporting

Please select the one below that is the best fit for your research. If you are not sure, read the appropriate sections before making your selection.

☐ Life sciences ☒ Behavioural & social sciences ☐ Ecological, evolutionary & environmental sciences

For a reference copy of the document with all sections, see nature.com/documents/nr-reporting-summary-flat.pdf

Behavioural & social sciences study design

All studies must disclose on these points even when the disclosure is negative.

Study description	Quantitative experimental
Research sample	89 participants completed the study, 40 patients with a DSM-IV diagnosis of schizophrenia and forty-nine healthy controls that were matched for age and sex. The patients were further subdivided with 16 classified as chronic cannabis users and 24 as non-users. The healthy controls were also subdivided with 22 classified as chronic cannabis users and 27 as non users. The samples are representative of the clinical syndromes studied and were chosen in order to study the effects of chronic cannabis use and schizophrenia on reward processing mechanisms of the brain.
Sampling strategy	Sampling of patients was based on diagnostic criteria from patients of the Athens University, First Psychiatry Clinic at Eginition Hospital. There was no randomization process to experimental groups. Controls were recruited from personnel of the Clinic and by word of mouth. Chronic cannabis users were recruited from the community with criteria to match in age and sex to the patient group. Sample sizes were not predetermined by calculation but numbers were chosen to approximate those of other similar published neuroimaging studies.
Data collection	Behavioural data were collected using special equipment for behavioural data collection inside an MR imaging device (Cedrus California USA) and were recorded in a behavioural acquisition PC. Neuroimaging data were recorded using the specific software of the Philips Achieva 3.0 Tesla TX MRI scanner. The researcher was present duirng data acquisition outside the MR room monitoring the behavioural responses of the participant. The researcher was not blind to the hypotheses tested and the experimental procedures but the experiment was fully computerized so that the experimenter did not intervene in the data collection process.
Timing	Data were collected from 2016 to 2018 (three year period).
Data exclusions	Behavioural data were excluded from 6 patients based on predefined exclusion criteria of performance that is at random level. Neuroimaging data were excluded from another 10 participants based on predefined criteria for preprocessing these data and excluding data that have low quality signals.
Non-participation	All 89 participants agreed to and participated in the study.
Randomization	There was no allocation of participants to experimental groups.

Reporting for specific materials, systems and methods

We require information from authors about some types of materials, experimental systems and methods used in many studies. Here, indicate whether each material, system or method listed is relevant to your study. If you are not sure if a list item applies to your research, read the appropriate section before selecting a response.

Materials & experimental systems

n/a	Involved in the study	
☐	☐	Antibodies
☐	☐	Eukaryotic cell lines
☐	☐	Palaeontology and archaeology
☐	☐	Animals and other organisms
☐	☒	Human research participants
☐	☐	Clinical data
☐	☐	Dual use research of concern

Methods

n/a	Involved in the study	
☐	☐	ChIP-seq
☐	☐	Flow cytometry
☐	☒	MRI-based neuroimaging

Antibodies

Antibodies used	*Describe all antibodies used in the study; as applicable, provide supplier name, catalog number, clone name, and lot number.*
Validation	*Describe the validation of each primary antibody for the species and application, noting any validation statements on the manufacturer's website, relevant citations, antibody profiles in online databases, or data provided in the manuscript.*

Eukaryotic cell lines

Policy information about cell lines

Cell line source(s)	*State the source of each cell line used.*
Authentication	*Describe the authentication procedures for each cell line used OR declare that none of the cell lines used were authenticated.*
Mycoplasma contamination	*Confirm that all cell lines tested negative for mycoplasma contamination OR describe the results of the testing for mycoplasma contamination OR declare that the cell lines were not tested for mycoplasma contamination.*
Commonly misidentified lines (See ICLAC register)	*Name any commonly misidentified cell lines used in the study and provide a rationale for their use.*

Palaeontology and Archaeology

Specimen provenance	*Provide provenance information for specimens and describe permits that were obtained for the work (including the name of the issuing authority, the date of issue, and any identifying information).*
Specimen deposition	*Indicate where the specimens have been deposited to permit free access by other researchers.*
Dating methods	*If new dates are provided, describe how they were obtained (e.g. collection, storage, sample pretreatment and measurement), where they were obtained (i.e. lab name), the calibration program and the protocol for quality assurance OR state that no new dates are provided.*

☐ Tick this box to confirm that the raw and calibrated dates are available in the paper or in Supplementary Information.

Ethics oversight	*Identify the organization(s) that approved or provided guidance on the study protocol, OR state that no ethical approval or guidance was required and explain why not.*

Note that full information on the approval of the study protocol must also be provided in the manuscript.

Animals and other organisms

Policy information about studies involving animals; ARRIVE guidelines recommended for reporting animal research

Laboratory animals	*For laboratory animals, report species, strain, sex and age OR state that the study did not involve laboratory animals.*
Wild animals	*Provide details on animals observed in or captured in the field; report species, sex and age where possible. Describe how animals were caught and transported and what happened to captive animals after the study (if killed, explain why and describe method; if released, say where and when) OR state that the study did not involve wild animals.*
Field-collected samples	*For laboratory work with field-collected samples, describe all relevant parameters such as housing, maintenance, temperature, photoperiod and end-of-experiment protocol OR state that the study did not involve samples collected from the field.*
Ethics oversight	*Identify the organization(s) that approved or provided guidance on the study protocol, OR state that no ethical approval or guidance was required and explain why not.*

Note that full information on the approval of the study protocol must also be provided in the manuscript.

Human research participants

Policy information about studies involving human research participants

Population characteristics	see above
Recruitment	see above
Ethics oversight	The study was approved by the ethics committee of the Eginition University Hospital.

Note that full information on the approval of the study protocol must also be provided in the manuscript.

Clinical data

Policy information about clinical studies

All manuscripts should comply with the ICMJE guidelines for publication of clinical research and a completed CONSORT checklist must be included with all submissions.

Clinical trial registration	*Provide the trial registration number from ClinicalTrials.gov or an equivalent agency.*
Study protocol	*Note where the full trial protocol can be accessed OR if not available, explain why.*

Data collection	*Describe the settings and locales of data collection, noting the time periods of recruitment and data collection.*
Outcomes	*Describe how you pre-defined primary and secondary outcome measures and how you assessed these measures.*

Dual use research of concern

Policy information about [dual use research of concern]

Hazards

Could the accidental, deliberate or reckless misuse of agents or technologies generated in the work, or the application of information presented in the manuscript, pose a threat to:

No	Yes	
☒	☐	Public health
☒	☐	National security
☒	☐	Crops and/or livestock
☒	☐	Ecosystems
☒	☐	Any other significant area

Experiments of concern

Does the work involve any of these experiments of concern:

No	Yes	
☒	☐	Demonstrate how to render a vaccine ineffective
☒	☐	Confer resistance to therapeutically useful antibiotics or antiviral agents
☒	☐	Enhance the virulence of a pathogen or render a nonpathogen virulent
☒	☐	Increase transmissibility of a pathogen
☒	☐	Alter the host range of a pathogen
☒	☐	Enable evasion of diagnostic/detection modalities
☒	☐	Enable the weaponization of a biological agent or toxin
☒	☐	Any other potentially harmful combination of experiments and agents

ChIP-seq

Data deposition

☐ Confirm that both raw and final processed data have been deposited in a public database such as GEO.

☐ Confirm that you have deposited or provided access to graph files (e.g. BED files) for the called peaks.

Data access links<br>*May remain private before publication.*	*For "Initial submission" or "Revised version" documents, provide reviewer access links. For your "Final submission" document, provide a link to the deposited data.*
Files in database submission	*Provide a list of all files available in the database submission.*
Genome browser session<br>(e.g. UCSC)	*Provide a link to an anonymized genome browser session for "Initial submission" and "Revised version" documents only, to enable peer review. Write "no longer applicable" for "Final submission" documents.*

Methodology

Replicates	*Describe the experimental replicates, specifying number, type and replicate agreement.*
Sequencing depth	*Describe the sequencing depth for each experiment, providing the total number of reads, uniquely mapped reads, length of reads and whether they were paired- or single-end.*
Antibodies	*Describe the antibodies used for the ChIP-seq experiments; as applicable, provide supplier name, catalog number, clone name, and lot number.*
Peak calling parameters	*Specify the command line program and parameters used for read mapping and peak calling, including the ChIP, control and index files used.*
Data quality	*Describe the methods used to ensure data quality in full detail, including how many peaks are at FDR 5% and above 5-fold enrichment.*

Software | *Describe the software used to collect and analyze the ChIP-seq data. For custom code that has been deposited into a community repository, provide accession details.*

Flow Cytometry

Plots

Confirm that:

- [ ] The axis labels state the marker and fluorochrome used (e.g. CD4-FITC).
- [ ] The axis scales are clearly visible. Include numbers along axes only for bottom left plot of group (a 'group' is an analysis of identical markers).
- [ ] All plots are contour plots with outliers or pseudocolor plots.
- [ ] A numerical value for number of cells or percentage (with statistics) is provided.

Methodology

Sample preparation	*Describe the sample preparation, detailing the biological source of the cells and any tissue processing steps used.*
Instrument	*Identify the instrument used for data collection, specifying make and model number.*
Software	*Describe the software used to collect and analyze the flow cytometry data. For custom code that has been deposited into a community repository, provide accession details.*
Cell population abundance	*Describe the abundance of the relevant cell populations within post-sort fractions, providing details on the purity of the samples and how it was determined.*
Gating strategy	*Describe the gating strategy used for all relevant experiments, specifying the preliminary FSC/SSC gates of the starting cell population, indicating where boundaries between "positive" and "negative" staining cell populations are defined.*

- [ ] Tick this box to confirm that a figure exemplifying the gating strategy is provided in the Supplementary Information.

Magnetic resonance imaging

Experimental design

Design type	task, event-related design
Design specifications	Each participant performed 6 blocks of 60 trials per block. Each trial lasted for a period of 4 or 6 or 8 seconds in a randomized sequence. The average time length of each block was 6 minutes (360 seconds).
Behavioral performance measures	Reaction time and percent of correct response was recorded for each experimental condition. A correct response rate of >70% was used to establish that subjects were performing the task at above chance performance level (a two choice response task with 50% probability of each response type).

Acquisition

Imaging type(s)	functional (task-fMRI), structural (T1)
Field strength	3 Tesla
Sequence & imaging parameters	fMRI: (Pulse sequence/imaging type = T2* gradient echo EPI; Field of view = 192×240mm2; Matrix size = 64×80; Slice thickness = 3mm; Orientation = axial, parallel to the AC-PC line; TR = 2000ms; TE = 30ms; Flip angle: 90 deg)<br>3D-T1: (Pulse sequence/imaging type = 3D T1 TFE weighted; Field of view = 240×240mm2; Matrix size = 240×240; Slice thickness = 1mm; Orientation = sagittal; TR = 9.9ms; TE = 4.6ms; Flip angle: 8 deg)
Area of acquisition	Whole-brain cerebral coverage , excluding cerebellum

Diffusion MRI [ ] Used [x] Not used

Preprocessing

Preprocessing software	SPM12 toolbox for MATLAB; fMRI module with the standard pipeline for realignment, unwarping, slice time correction (with reference slice: middle slice), co-registration, segmentation and normalization, and smoothing (with an 8mm full width at half maximum (FWHM) Gaussian kernel).
Normalization	Normalization using affine registration with the tissue probability maps combined with regularization (ICBM space template – European brains)

Normalization template	ICBM152 (MNI) - European brains
Noise and artifact removal	Quality control was performed using ArtRepair software (Center for Interdisciplinary Brain Sciences, Stanford University, USA). Data with registered motion >3 mm or 1 degree was excluded, in keeping with the general rule for exclusion of data with motion greater than the dimensions of a single voxel (Soares et al., 2016). Following the pre-processing stage, high-pass filtering of 128 s cut-off was applied to the voxel time-series to remove low-pass physiological components such as respiration and heartbeat.
Volume censoring	ArtRepair software (see above).

Statistical modeling & inference

Model type and settings	A first-level within-subject analysis was carried out and GLM was applied to the images from each participant. Apart from the regressors of the main model, additional regressors included motion correction parameters estimated from the realignment step of the pre-processing. T-contrasts were calculated to measure the contrasts of interest, as described in detail in the manuscript. A second-level analysis was carried out using full-factorial model of the SPM12.
Effect(s) tested	Main effect, 2-way and 3-way interactions.

Specify type of analysis: ☐ Whole brain ☒ ROI-based ☐ Both

Anatomical location(s)	ROI-based analysis focused on striatum (NAcc, caudate and putamen), thalamus, amygdala and insula (dorsal and ventral anterior, posterior). Striatum, thalamus and amygdala were defined structurally using the Automated Anatomical Labelling atlas 3 (AAL3). Using mean MNI coordinates from a prior study (Deen et al., 2011), the insular sub-regions were manually defined on T1 (as previously reported, Moran et al., 2013) in order to ensure the inclusion of all anatomically relevant regions and the exclusion of anatomically irrelevant regions. All ROIs were defined in MNI space for both right and left hemispheres and were defined before any data analysis in order to avoid bias.
Statistic type for inference (See Eklund et al. 2016)	Small volume correction (SVC) of sphere with 10mm radius surrounding the peak voxel was applied within the ROIs and clusters were considered significant if the family-wise error (FWE) corrected peak p-value was significant at $p < 0.05$.
Correction	The analyses were restricted to the previous ROIs for which control for multiple comparisons was performed using Gaussian random field (GRF) theory for small volume. Small volume correction (SVC) of sphere with 10mm radius surrounding the peak voxel was applied within these regions and clusters were considered significant if the family-wise error (FWE) corrected peak p-value was significant at $p < 0.05$, as in previous studies. A minimum cluster size threshold of 3 contiguous voxels was considered in all analyses to avoid type-1 errors.

Models & analysis

n/a	Involved in the study
☒	☐ Functional and/or effective connectivity
☒	☐ Graph analysis
☒	☐ Multivariate modeling or predictive analysis
